# The revised German guideline on the monitoring for intakes of radionuclides

**DOI:** 10.1007/s00411-025-01183-7

**Published:** 2025-12-18

**Authors:** Oliver Meisenberg, Simone Löscher, Thomas van Appeldorn, Bastian Breustedt, Martina Froning, Daniel Gehre, Augusto Giussani, Heribert Hänscheid, Ulrich Kratzel, Rolf Michel, Clemens Scholl, Carsten Wanke, Astrid Imig

**Affiliations:** 1 Federal Office for Radiation Protection, Medical and Occupational Radiation Protection, Ingolstädter Landstr. 1, 85764 Oberschleißheim, Germany; 2 German-Swiss Association for Radiation Protection, Jülich, Germany; 3 PreussenElektra GmbH, Isar Nuclear Power Plant, Essenbach, Germany; 4 Karlsruhe Institute of Technology, Karlsruhe, Germany; 5 Forschungszentrum Jülich, Jülich, Germany; 6 Technische Universität Dresden, Dresden, Germany; 7 German Standardisation Committee on Radiology, Working Group NA 080-00-03 AA Nuclear Medicine, Berlin, Germany; 8 Department of Nuclear Medicine, University Hospital Würzburg, Würzburg, Germany; 9 Bavarian Environment Agency, Kulmbach, Germany; 10 Landesamt für Gesundheit und Arbeitsschutz Nordrhein-Westfalen, Düsseldorf, Germany; 11 Hannover Medical School, Hannover, Germany; 12 Federal Ministry for the Environment, Climate Action, Nature Conservation and Nuclear Safety, Bonn, Germany

**Keywords:** Internal monitoring, Bioassay, Incorporation, Legislation, Dosimetry

## Abstract

In 2025, the guideline for the realisation of monitoring of occupational intakes of radionuclides (internal monitoring) in Germany was revised. For this purpose, an expert workgroup was mandated to consider the amended German radiation protection legislation, amended international standards and recommendations as well as other recent developments in the subject area. The methodology of the revised guideline for the calculation of the likely committed effective dose based on the handled activity and incorporation factors adapts international recommendations and standards. However, the definitions and values of the subfactors from which the incorporation factors are calculated deviate from international recommendations, for example the physical form safety factor considers the volume or mass of the handled radioactive material. For applications in nuclear medicine, specific incorporation factors based on recent literature are tabulated. Regarding the monitoring of persons of childbearing potential, an assessment of the equivalent dose for the uterus and if required a monthly monitoring are prescribed. Quality assurance of approved monitoring services is based optionally on accreditation or on audits by public authorities. For workplace monitoring, technical requirements, in particular quality-assurance procedures, are also specified. Regarding the dose assessment, a reference method and several individual methods are described, with the investigation threshold for changing to individual methods corresponding to an effective dose of 6 mSv during the calendar year. For the reference method, standard assumptions (inhalation, AMAD 5 μm for workplaces, 1 μm for emergency workers with environmental exposure) are applied.

## Introduction

The monitoring of individuals for occupational intakes of radionuclides (internal monitoring) is the repeated or one-time measurement of the exposure of persons to radioactive substances in order to assess the intakes of radionuclides into their bodies and to calculate the committed internal radiation doses caused by these intakes. It comprises methods of in-vivo monitoring (measurements with whole-body and partial-body counters), in-vitro measurements of biological samples (urine and faeces), both summarised as bioassay, and workplace monitoring (measurements of the concentration of airborne radionuclides at the workplace). The guideline that has existed for a harmonised realisation of internal monitoring in Germany since 2007 (BMU 2007) was revised in 2025 because of the following reasons:

- an amended radiation protection legislation (StrlSchG 2017; StrlSchV 2018) based on the Euratom Basic Safety Standards (Council of the European Union 2013),
- new or amended international recommendations and standards such as the European Commission (EC) Technical Recommendations (Etherington et al. 2018), IDEAS Guidelines (Castellani et al. 2013), ISO 16637 (ISO 2016), ISO 20553 (ISO 2025) or, in particular for dose assessment, ISO 27048 (ISO 2011),
- new models for the biokinetic behaviour of radionuclides in the human body, leading to modified dose coefficients and values of retention and excretion (ICRP 2015),
- new methods in various fields of the application of radioactive substances, in particular in nuclear medicine, and radionuclides that have recently gained relevance,
- increased experience in the realisation of internal monitoring.

The guideline was officially published in the Joint Ministerial Bulletin (GMBl 2025) and is available together with explanations and accompanying documents on the website of the German Federal Office for Radiation Protection (BfS 2025).

The previous as well as the revised guideline address to competent authorities, internal monitoring services and employers whose workers handle unsealed radioactive material. They contain prescriptions regarding:

- the requirement of routine internal monitoring based on the likely committed effective dose,
- the calculation of the likely committed effective dose,
- the assignment of responsibilities to the employers, internal monitoring services and public authorities,
- requirements for the realisation of routine measurements such as their frequency or precision,
- organisational requirements for the internal monitoring services such as those regarding their quality-assurance programme,
- the calculation of the intake, committed equivalent doses and the committed effective dose and its influencing parameters and assumptions.

In contrast to the EC Technical Recommendations, neither cases of cutaneous and wound contamination (regarding contamination measurements and the calculation of the local skin dose) nor exposure to radon and its progeny are covered by the guideline.

For the purpose of revising the existing guideline, a workgroup of 13 experts from public radiation protection authorities, internal monitoring services, nuclear medical departments and nuclear technology enterprises was formed in 2020. The draft was discussed with further experts from the same fields. Finally, it was approved by the Federal States Committee for Nuclear Energy and its Technical Committee for Radiation Protection.

In the following, several aspects of the revised guideline are presented and compared to international recommendations and standards, in particular to the EC Technical Recommendations (Etherington et al. 2018). The applied terminology is based on the Technical Recommendations.

## The system of internal monitoring of occupational exposure in Germany

Monitoring methods comprise in-vivo measurements, in-vitro measurements and workplace monitoring. Whereas the first two methods are applied by approved monitoring services (in German *behördlich bestimmte Messstellen*), the latter one is applied by the employer. The monitoring service or, in case of workplace monitoring, the employer is also responsible for the dose assessment and the data transfer to the national dose register. Workplace monitoring is accompanied by a yearly in-vivo or in-vitro measurement at an approved monitoring service as a validation of the measurement results at a random point in time *(verifizierende Messung)*.

Internal monitoring services (also called internal dosimetry services) are approved by competent public authorities, mainly regional radiation protection authorities of the German states. As of July 2025, 22 facilities are approved as internal monitoring services, run by public authorities, research centres, hospitals and nuclear enterprises. 12 of them provide in-vivo monitoring, 5 provide in-vitro monitoring of selected radionuclides and 5 provide both types of methods. The number of conducted measurements ranges among the services from below 10 to more than 1000 per year.

If the internal exposure is probably below the threshold for routine monitoring but not obviously negligible, so-called threshold measurements *(Schwellenwertmessungen)* are required. This is similar to confirmatory and triage monitoring according to the EC Technical Recommendations and is meant to prove that the threshold for routine monitoring is not exceeded. Threshold measurements are typically realised at the workplace by contamination measurements, workplace measurements of the airborne concentration of radionuclides or in-plant whole-body or partial-body measurements. Dose assessments must not be conducted using the results of these measurements and there is no need to conduct threshold measurements individually for every worker. There is no general requirement to validate the results of threshold measurements by random measurements at an approved monitoring service but such validation may be prescribed by the competent authority.

Beside routine monitoring *(regelmäßige Inkorporationsüberwachung)*, internal monitoring can also be carried out as special monitoring (after incidents, *Inkorporationsüberwachung aus besonderem Anlass*) and task-related monitoring (for the handling of radionuclides over a period of time that is shorter than one monitoring interval, *aufgabenbezogene Inkorporationsüberwachung*). The differentiation between these monitoring programmes depends only on the exposure conditions, not on the committed effective dose.

## Requirement of routine internal monitoring based on the likely committed effective dose

German radiation protection legislation prescribes a monitoring of the effective dose for all persons entering a supervised or controlled area unless it can be expected that the effective dose due to internal and external exposure during the calendar year falls below a threshold of 1 mSv (monitoring threshold, *Erfordernisschwelle*). If monitoring is required, there is only an immediate legal requirement to monitor the personal dose equivalent due to *external* exposure. Monitoring of the committed effective dose due to *internal* exposures can be imposed on the employer due to the conditions of exposure by the competent authority. Therefore, the guideline specifies the requirement of routine internal monitoring based on a combination of the likely effective dose due to total (external and internal) exposure and the contribution of the likely committed effective dose due to internal exposure. Routine internal monitoring of individuals is required

- if the likely committed effective dose due to internal exposure exceeds the monitoring threshold of 1 mSv per year or
- if the dose from external exposure remains reliably below 1 mSv and is therefore not monitored but the total effective dose may exceed 1 mSv due to an additional internal exposure that exceeds a threshold of 0.5 mSv per year. This is similar to the reduction of the monitoring threshold by a factor of 2 that was considered in ISO 20553 before 2025 and which has been replaced by a subtraction of the potential dose due to external exposure (ISO 2006, 2025).

In any other case where the likely committed effective dose due to internal exposure exceeds a value of 0.5 mSv per year, threshold measurements are required. Below this value, no measures of internal monitoring are required.

## Calculation of the likely committed effective dose based on the handled activity and incorporation factors

The likely committed effective dose due to internal exposure, called potential dose *E*_pot_ in the guideline (*potenzielle effektive Dosis)*, is applied to decide whether individual internal monitoring shall be performed. Its general definition according to Eq. 1 conforms to ISO 16637.

1 $$\:{E}_{\mathrm{pot}}={\sum}_{i}{a}_{i}\cdot\:{A}_{i}\cdot\:{e}_{i}$$

where:

*i* running index for work processes, which are defined by the radionuclide, handled activity and the incorporation factor,

*a*_i_ fraction of the handled activity that can be incorporated (incorporation factor) for work process *i*,

*A*_i_ activity handled during the calendar year (cumulative activity) for work process *i*,

*e*_i_ effective dose coefficient for the respective radionuclide in work process *i*.

*E*_pot_ corresponds to the decision factor *D* that was originally introduced by IAEA and that was further discussed in the EC Technical Recommendations in units of mSv.

The activity handled during the calendar year is the sum of the activities that are handled in each single execution of the work process over the calendar year. It can also be calculated as the product of the average single handled activity and the number of executions of the work process or as the total activity put into process over the calendar year.

Two types of handling are distinguished: elementary and complex handling, where elementary handling refers to a single step of work and complex handling refers to a process of several subsequent steps. Several simple handlings can be combined to one complex handling in order to facilitate the calculation.

Incorporation factors are described in a variety of publications. Typical orders of magnitude are 10^− 6^ for handling under fume hoods and 10^− 5^ for handling without protective measures (Brodsky 1977, 1980).

The guideline adopts the general calculation of the incorporation factor as the product of three single factors as applied both by IAEA RS-G-1.2 and ISO 16637:

- physical form safety factor *f*_fs_
- handling safety factor *f*_hs_
- protection safety factor *f*_ps_

*f*_fs_ considers the influence of “the physical and chemical properties of the material being handled” on the incorporation risk. IAEA RS-G-1.2 and ISO 16637 assign it a value of 10^− 2^ for “the majority of cases” and of 10^− 3^ for “some cases, where it can be shown to be justified”, without detailing the nature of these cases or giving examples. The guideline elaborates on the influence of the physical form and defines the values of *f*_fs_ depending on the mass (for solids) or volume (for liquids and gases) of the radioactive material, considering the reduced risk of incorporating the same fraction of a material for larger masses and volumes (Table 1).

ISO 16637 recognises that IAEA RS-G-1.2 contains incorporation factors that are several orders of magnitude too high and tries to correct this deviation by introducing a fixed correction factor *f*_intake_ of 10^− 4^. In contrast to this approach, the guideline corrects the deviation by amending the values of *f*_fs_ and defining them between 10^− 6^ and 10^− 4^. Additionally, the factors *f*_workload_ and *f*_handled activity_ introduced in ISO 16637 are omitted in the guideline because the handled activity as defined in the guideline is that of the individual worker, not for the workplace.

*f*_hs_ considers different types of handling. In contrast to ISO 16637, the smallest value is not attributed to “storage (stock solution)” as a persisting state but to several processes related to storing in order to consistently relate to processes of handling. Several of the other types of handling are defined differently to allow for the described distinction between elementary and complex types (Table 2).

*f*_ps_ adopts the effect of laboratory safety equipment from ISO 16637 but adds the beneficial effect of adequate ventilation of the laboratory with an additional factor of 0.5 (Table 3).

For typical types of handling, this methodology yields an incorporation factor of 10^− 5^ without protective measures (*f*_fs_=10^− 5^, *f*_hs_=1, *f*_ps_=1) but makes it possible to consider various working conditions that lead to different risks of intakes. For established work processes in nuclear medicine, specific incorporation factors based on practical experience (Happel et al. 2017; Wanke et al. 2022) are tabulated (Table 4). All of them are equal to or smaller (up to two orders of magnitude) than the respective generic incorporation factor calculated from *f*_fs_, *f*_hs_ and *f*_ps_,. If knowledge about incorporation factors for the respective work process at the respective workplace based on measurements exists, the use of these factors shall be preferred to that of the generic factors.

**Table 1:** Physical form safety factor *f*_fs_

Volume (ml) or mass (g)	f_fs_
> 100	10^− 6^
1–100	10^− 5^
< 1	10^− 4^

**Table 2:** Handling safety factor *f*_hs_

Type of handling	f_hs_
In-plant transport, packing, unpacking, transferring to storage, loading^1^	0.01
Elementary handling without increased risk of release	0.1
Elementary handling with increased risk of release or complex handling without increased risk of release	1
Complex handling with increased risk of release	10
Handling of volatile or dusty substances	100

This type of handling need not be considered if the radioactive material is enclosed in a tight container whose surface was checked for contamination

**Table 3:** Protection safety factor *f*_ps_

Protective measure	f_ps_
Handling without protective measures	1
Handling under fume hood	0.1
Radioactive substance in closed container (tight receptacle, glove box)	0.01
Additional factor for handling in a room with at least 25 m^3^/h exhaust airstream per m² of useable surface	0.5

**Table 4:** Examples of tabulated specific incorporation factors for applications in nuclear medicine

Application	Generic value of			Generic incorporation factor	Specific incorporation factor based on literature review
	*f*_fs_	*f*_hs_	*f*_ps_		
Elution from approved radionuclide generators	10^− 5^	0.1	0.5	5·10^− 7^	10^− 7^
Scintigraphy or PET imaging (excluding administration of radiopharmaceuticals)	10^− 6^	0.1	0.5	5·10^− 8^	0
Production of radionuclide aerosols and gases from generators	10^− 4^	100	1	10^− 2^	10^− 4^
Scintigraphy or PET imaging with aerosols and gases (excluding administration of radiopharmaceuticals)	10^− 4^	100	0.1	10^− 3^	10^− 5^
Administration of therapeutic radiopharmaceuticals (except of volatile iodine compounds)	10^− 5^	0.1	0.5	5·10^− 7^	10^− 7^
Administration of therapeutic radiopharmaceuticals (volatile iodine compounds)	10^− 5^	100	0.5	5·10^− 4^	5·10^− 4^
Automated labelling of pharmaceuticals in glove boxes	10^− 4^	0.1	0.01	10^− 7^	10^− 7^

## Monitoring of persons of childbearing potential

German radiation protection legislation defines a limit of the equivalent dose for the uterus of 2 mSv per month for persons of childbearing potential. Therefore, the guideline provides an additional monitoring threshold of 0.5 mSv regarding the likely committed equivalent dose for the uterus per month for these persons. The likely committed equivalent dose is calculated analogously to the likely committed effective dose, applying the respective dose coefficient for the uterus and considering the activity that is handled during one month instead of one year. If this additional monitoring threshold is exceeded, internal monitoring of persons of childbearing potential must be conducted at least monthly. In any case, the equivalent dose for the uterus must be calculated and reported to the national dose register by the monitoring service. No provision is contained under which conditions, in particular at which age, a person shall be considered as having a potential of childbearing because such provision must not be issued on the level of a guideline but in the underlying legislation. However, such provision is not contained in relevant German legislation either.

## Monitoring of the exposure of unborn children

For pregnant persons starting from the point in time when the pregnancy is reported to the employer, the working conditions must be organised in such way that an occupational internal exposure of the pregnant person is excluded. However, unlikely events still can make an intake of radionuclides by the pregnant person possible. An additional dose limit to the unborn child of 1 mSv applies, including all exposures of the unborn child (external exposure of the pregnant person, external exposure of the unborn child due to radionuclides in the body of the pregnant person and internal exposure due to radionuclides in the body of the child during the pregnancy and beyond birth). The latter two contributions are monitored within the system of internal monitoring of the pregnant person. For the calculation of the effective dose of the unborn child from the latter two contributions, dose coefficients were published for different times of intake with regard to the time of conception (BfS 2022).

## Monitoring intervals

Similar to the EC Technical Recommendations, the monitoring interval of routine monitoring is determined from two criteria: the factor-3 criterion and the 1 mSv criterion. The first criterion assures that under the standard assumption of an intake at the midpoint of the monitoring interval the committed effective dose is underestimated by a factor of not greater than 3 as compared to an intake at the beginning of the monitoring interval. The second criterion assures that over the sum of all measurements in a year and assuming intakes at the beginnings of the monitoring intervals a committed effective dose of 1 mSv can be detected. The second criterion is dependent on the individual detection limit that can be achieved by the internal monitoring service. No criterion is introduced to avoid a possible overestimation of the committed effective dose although this might be desirable (Meisenberg and Mohsin 2025). At least 2 measurements per year are mandatory. For a variety of radionuclides, chemical forms and measurement methods, monitoring intervals are tabulated under three assumptions: the typical detection limit of the respective method, the longest monitoring interval possible (related to the smallest detection limit), and the highest detection limit possible (related to the shortest monitoring interval).

## Requirements regarding the quality-assurance of internal monitoring services and of workplace monitoring

The previous guideline required internal monitoring services to be accredited according to ISO/IEC 17025 (ISO 2017). This has proved difficult to implement by smaller monitoring services, which are, however, essential to guarantee a dense network throughout Germany. Thus, this requirement is now supplemented by the alternative option of having the monitoring service audited by the Federal Office for Radiation Protection or the competent authority. For these audits, a list of checkpoints partly on a yearly basis and partly on a 5-year basis, which are tailored to the requirements of internal monitoring services, is provided. For example, they consider the specific metrological conditions and reporting duties of internal monitoring services. In any case, approved monitoring services shall participate in the yearly proficiency tests and internal dose-assessment exercises, the so-called dosimetric case studies, offered by the Federal Office for Radiation Protection.

Measurements of the airborne radionuclide concentration at the workplace that are used for individual dose assessments must also be subject to quality assurance. These measures comprise visual inspections of the samplers and measurement devices as well as half-yearly checks of sampling installations, detectors, electronics and warning signals using a radioactive check source.

## Dose assessment

The guideline distinguishes between a reference method of dose assessment and several more specific individual methods, comparable to the distinction between routine and special dose assessment in the EC Technical Recommendations. All of these methods relate to routine monitoring, special monitoring after incidents, and task-related monitoring. In the reference method *(Referenzverfahren)*, in accordance with ISO 27048, Clause 7.1.3, an intake via inhalation, an AMAD of 5 μm (1 μm for emergency workers when exposed to environmental radioactivity) and an acute intake at the midpoint of the monitoring interval (except of special monitoring after incidents, where the time of intake is known) are assumed.

For an individual dose assessment *(Individualverfahren)*, three methods are described. They comprise an assessment based on individual information on the conditions of exposure, an adjustment of the biokinetic model and an integration of the measured activity over time, the last two being based on the results of repeated measurements.

Both in the reference and in the individual methods, a correction of the measurement results is required to consider the remaining contribution of earlier intakes. In contrast to ISO 27048, Clause 7.1.2.2, a new significant intake is assumed if the corrected activity exceeds the decision threshold of the measurement method; scattering factors are not applied in this step. An earlier intake needs not be considered any more if disregarding it leads to an overestimation of the calculated committed effective dose by less than 0.05 mSv (termination criterion).

Individual methods shall be applied if the prescribed monitoring interval was disregarded or if the investigation threshold (effective dose of 6 mSv during the calendar year for routine monitoring; *Nachforschungsschwelle*) is exceeded, in the latter case for all measurements during the calendar year. For special monitoring after a suspected incident, the threshold is 1 mSv instead of 6 mSv and applies to an individual assessment only of the single measurement. Besides, a transition to an individual assessment is always possible on a voluntary basis if the employer, competent authority and monitoring service agree. In contrast to the EC Technical Recommendations, further criteria for the application of individual methods such as unexpected exposures or intakes that give rise to the possibility of a further exceedance of the dose limit in the remainder of the calendar year are not compulsory. This is because German radiation protection legislation requires the employers to provide information about the conditions of exposure only in the case of a potential exceedance of the dose limit; in other cases, there is no legal basis for a dosimetry in which the specific exposure conditions are considered. For a dose limit of 20 mSv in the calendar year, this potential is identified with an exceedance of the investigation threshold of 6 mSv after application of the reference method. Besides, the other criteria for the application of individual methods were regarded as unjustifiably strict. In contrast to the IDEAS Guidelines (Castellani et al. 2013), a complex distinction of cases (called stages) was not considered for reasons of practicability.

## Calculation of characteristic limits of measurement results

Internal monitoring services are required to calculate and report the uncertainty and decision threshold of the activity for each measurement. Besides, they need to calculate the detection limit in order to assess if their methods are sufficiently sensitive. These calculations shall be conducted based on ISO 11929 (ISO 2019), with probabilities α and β for an error of first and second kind of 5%. Uncertainties of type A and of type B shall be considered.

## Conclusion

The revision of the German guideline on the monitoring for intakes of radionuclides was intended to extensively adopt international recommendations. However, it was found that several adaptations and concretisations had to be introduced in order to provide a better and more precise application by its users. Most considerably, this affected the specification of incorporation factors, for which only little primary literature and several different and deviating statements in international recommendations exist. Other provisions such as a compulsory calculation of the effective dose from case-specific parameters in cases other than a possible exceedance of the dose limit could not be adopted due to national legal restrictions. Requirements for the work of internal monitoring services such as those concerning their quality assurance had to be adjusted for reasons of practicality and cooperativeness of the monitoring services. All aspects considered, the revised guideline is believed to provide a practicable basis for internal monitoring.

The presented aspects of the guideline are meant as a contribution to the discussion of further amendments of existing international recommendations and standards. Beyond the scope of the guideline, further standardisation is deemed necessary, in particular regarding technical aspects of the monitoring of the concentration of airborne radionuclides at the workplace.

## Data Availability

No datasets were generated or analysed during the current study.
